# C9orf72 plays a central role in Rab GTPase-dependent regulation of autophagy

**DOI:** 10.1080/21541248.2016.1240495

**Published:** 2016-10-21

**Authors:** Christopher P. Webster, Emma F. Smith, Andrew J. Grierson, Kurt J. De Vos

**Affiliations:** Sheffield Institute for Translational Neuroscience (SITraN), Department of Neuroscience, University of Sheffield, Sheffield, UK

**Keywords:** ALS, autophagy, C9orf72, FTD, Rab GTPase, ULK1

## Abstract

A GGGGCC hexanucleotide repeat expansion in the first intron of the *C9orf72* gene is the most common genetic defect associated with amyotrophic lateral sclerosis (ALS) and frontotemporal dementia (FTD) (C9ALS/FTD). Haploinsufficiency and a resulting loss of C9orf72 protein function has been suggested as a possible pathogenic mechanism in C9ALS/FTD. C9ALS/FTD patients exhibit specific ubiquitin and p62/sequestosome-1 positive but TDP-43 negative inclusions in the cerebellum and hippocampus, indicating possible autophagy deficits in these patients. In a recent study, we investigated this possibility by reducing expression of C9orf72 in cell lines and primary neurons and found that C9orf72 regulates the initiation of autophagy. C9orf72 interacts with Rab1a, preferentially in its GTP-bound state, as well as the ULK1 autophagy initiation complex. As an effector of Rab1a, C9orf72 controls the Rab1a-dependent trafficking of the ULK1 initiation complex prior to autophagosome formation. In line with this function, C9orf72 depletion in cell lines and primary neurons caused the accumulation of p62/sequestosome-1-positive inclusions. In support of a role in disease pathogenesis, C9ALS/FTD patient-derived iNeurons showed markedly reduced levels of autophagy. In this Commentary we summarise recent findings supporting the key role of C9orf72 in Rab GTPase-dependent regulation of autophagy and discuss autophagy dysregulation as a pathogenic mechanism in ALS/FTD.

## Introduction

Amyotrophic lateral sclerosis (ALS) is the most common adult onset motor neuron disorder. ALS is caused by selective degeneration of upper and lower motor neurons, which leads to progressive muscle weakness, gait abnormalities, paralysis and ultimately death. Frontotemporal dementia (FTD) is the second most common form of dementia in the under 65s. FTD is characterized by loss of neurons predominantly in the frontal and temporal cortex, leading to changes in personality, behavior and cognitive function. ALS and FTD are now recognized to be at the extremes of a spectrum of the same disorder with up to 25% of ALS cases clinically diagnosed with FTD and approximately 50% of FTD cases displaying some degree of motor neuron involvement.^1^

A GGGGCC hexanucleotide repeat expansion within the first intron of the *C9orf72* gene was found to be the most common genetic defect associated with both ALS and FTD (referred to as C9ALS/FTD).^2,3^ The pathogenic mechanism by which the repeat expansion causes disease may involve gain-of-toxic-function mechanisms, namely RNA toxicity^2,4-8^ and protein toxicity by aberrant dipeptide repeat protein (DPR) accumulation.^9-13^ Alternatively, reduced C9orf72 mRNA levels in a range of patient tissues and patient-derived cell lines^2,14-18^ and reduced C9orf72 protein levels in the frontal cortex of C9ALS/FTD patients^19,20^ suggest loss-of-function by *C9orf72* haploinsufficiency may also contribute to C9ALS/FTD. Mild neurodegeneration and cognitive impairment have been reported in some gain-of-toxic-function GGGGCC repeat C9orf72 BAC-transgenic mice^21,22^ but not others,^23,24^ while loss-of-function *C9orf72* knockout mice exhibit an immunological rather than neuronal phenotype.^25-29^ Thus neither loss- nor gain-of-function appears sufficient to model all aspects of disease *in vivo*, suggesting that all 3 mechanisms might be conspiring to cause C9ALS/FTD.

The alternatively spliced *C9orf72* gene codes for 2 protein isoforms, a 481 amino acid (aa) “long” isoform (C9orf72L) and a 222 aa “short” isoform (C9orf72S). C9orf72 does not show obvious sequence homology to other proteins, but bioinformatics analysis revealed that the C9orf72 protein is structurally related to Differentially Expressed in Normal and Neoplasia (DENN) domain-containing proteins, which function as GDP/GTP exchange factors (GEFs) for the Rab family of GTPases.^30,31^ Rab GTPases function as molecular switches that alternate between a GTP-bound active state and a GDP-bound inactive state. GEFs catalyze the exchange of GDP for GTP while GTPase activating proteins (GAPs) catalyze GTP hydrolysis to GDP. GTP-bound, active Rabs recruit and/or activate a variety of effector molecules and in doing so Rabs control a range of cellular trafficking events, including autophagy (reviewed in refs. 32, 33).

**Figure 1.**
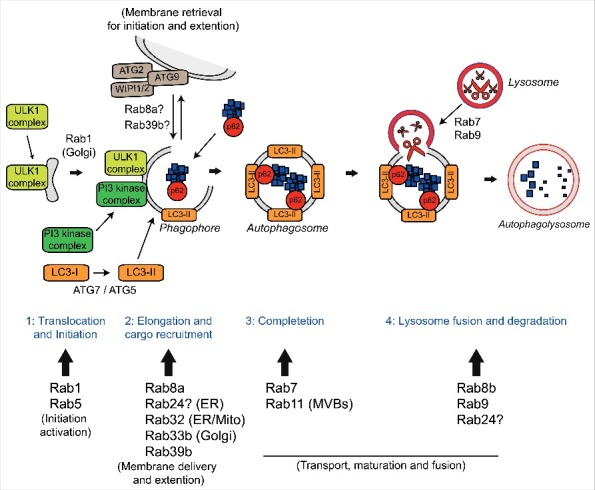
Autophagy and the Rab GTPases. The four stages of autophagy are indicated with the different Rabs involved at each stage. 1) Translocation of the ULK1 initiation complex to the phagophore is the first step in autophagy initiation. Rab1a mediates trafficking of the ULK1 complex to the phagophore and is involved in delivery of ATG9 positive membranes to the site of phagophore formation. Rab5 is involved in translocation of the Class III PI3 kinase complex and delivery of LC3-II. 2) Elongation of the phagophore membrane requires the Class III PI3 kinase complex. Rab8a, Rab24, Rab32, Rab33b and Rab39b are all involved in autophagosome formation and may aid in elongation by the delivery of additional membrane via ATG9/ATG2/WIPI1/2. Autophagy substrates are recruited to the growing phagophore by autophagy receptors such as p62/sequestosome-1 and optineurin. Autophagy receptors bind ubiquitin on the substrates and LC3-II on the nascent phagophore resulting in substrate delivery. 3) After completion and closure, autophagosomes are transported to allow fusion with the lysosome. Rab7 is involved in autophagosome transport while Rab11 delivers multi-vesicular bodies (MVBs) to the autophagosome, which appears to be required for maturation. 4) Autophagosome fusion with the lysosome allows degradation of the autophagic substrates. Rab7, Rab8b and Rab9 are involved in the fusion of autophagosomes and lysosomes, a process that may also require Rab24. Finally, autophagic substrates are degraded by the acid hydolases of the lysosome and recycled back to the cytoplasm.

Autophagy (derived from the Greek and meaning “self-eating”) is a highly conserved catabolic process that involves the delivery of cytosolic components to the lysosome for degradation. Macroautophagy, herein referred to as autophagy, requires the formation of a double membrane structure called the autophagosome, which encapsulates cytosolic substrates such as misfolded proteins and damaged organelles, before fusing with the lysosome to allow bulk degradation and recycling. The autophagy process can be subdivided into 4 steps, (i) translocation and initiation, (ii) elongation and cargo recruitment, (iii) completion, and (iv) lysosome fusion and degradation (reviewed in ref. 34). A large number of Rab GTPases have been shown to be involved at each stage of autophagy (reviewed in ref. 32) (Fig. 1).

Here we summarise the role of C9orf72 in Rab-mediated regulation of autophagy and discuss autophagy dysregulation as a pathogenic mechanism in ALS/FTD.

### C9orf72 and autophagy induction

While the pathology associated with C9ALS/FTD is in many ways consistent with other forms of ALS/FTD, including the classical TDP-43 neuronal cytoplasmic inclusions, C9ALS/FTD cases are distinguished by the presence of specific ubiquitin and p62/sequestosome-1 positive, TDP-43 negative, neuronal cytoplasmic inclusions in the cerebellum, hippocampus and neocortex.^16,35,36^ p62/sequestosome-1 is a well-characterized autophagy receptor that binds ubiquitinated substrates and recruits them to the nascent phagosome via interaction with phagosomal LC3-II (Fig. 1). Because p62/sequestosome-1 is itself degraded by autophagy in the process of delivering substrates, accumulations of p62/sequestosome-1 are indicative of defective autophagy.^37^ Thus, the presence of p62/sequestosome-1-positive inclusions together with *C9orf72* haploinsufficiency suggested the possibility of defective autophagy by loss of C9orf72 function in C9ALS/FTD.

To test for a role of C9orf72 in autophagy we depleted C9orf72 or, conversely, overexpressed C9orf72 in cell lines and primary neurons and quantified autophagy by monitoring LC3-II flux.^38^ We found that loss of C9orf72 disrupted autophagy at the initiation stage whereas overexpression of C9orf72 induced autophagy.^39^ These results corroborated other reports showing that loss of C9orf72 led to a disruption in autophagy in neuronal cell lines^40^ and *in vivo* in *C9orf72* knockout mice.^23,28^

The initiation of autophagy is controlled by the ULK1 autophagy initiation complex, which is comprised of Unc-51-like kinase 1 (ULK1), FAK family kinase-interacting protein of 200 kDa (FIP200), autophagy-related 13 (ATG13) and ATG101.^41-44^ When we disrupted the ULK1 complex by depletion of FIP200, C9orf72 overexpression was no longer able to induce autophagy indicating that C9orf72 acted at the level of the ULK1 complex.^39^ Using immunoprecipitation, *in vitro* binding and proximity ligation assays we went on to show a direct interaction between C9orf72 and ULK1, FIP200 and ATG13.^39^ The interaction with FIP200 confirmed a previously reported proteomics screen.^45^ In support of our findings, C9orf72, in complex with SMCR8 and WDR41, was independently reported to interact with the ULK1 initiation complex.^28^

**Figure 2.**
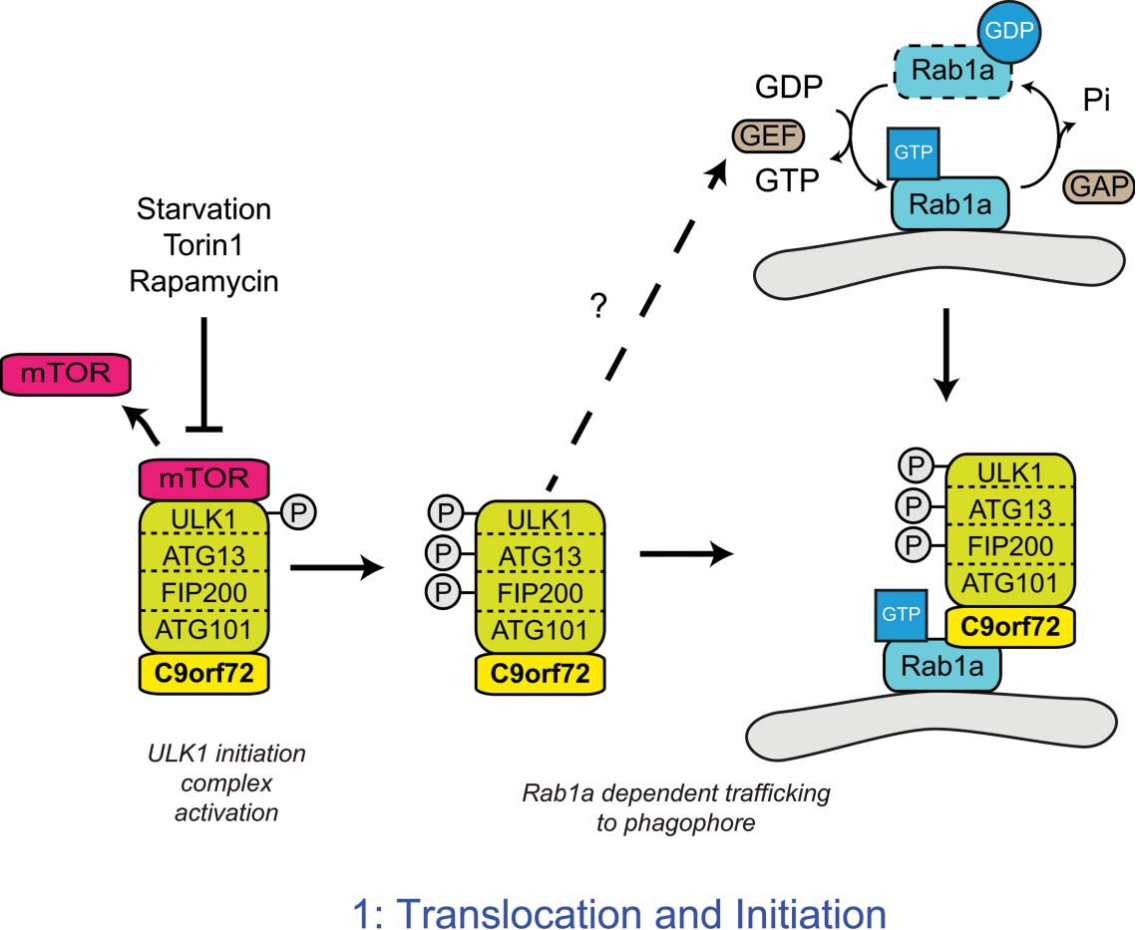
C9orf72 regulates the Rab1a dependent trafficking of the ULK1 complex. Upon inhibition of mTOR, the inhibitory phosphorylation of ULK1 is lost, leading to the activation of ULK1. Active ULK1 phosphorylates the other members of the initiation complex, including FIP200 and ATG13. Functioning as a Rab1a effector, C9orf72 mediates the interaction between the active ULK1 initiation complex and GTP-Rab1a within its target membrane. Thus C9orf72 controls the Rab1a dependent trafficking of the ULK1 initiation complex to the phagophore. How Rab1 is activated in response to autophagy induction is not yet known.

The ULK1 complex is kept inactive by mTOR, which phosphorylates ULK1 at serine 757.^46^ Inactivation of mTOR leads to its release from the ULK1 complex and ULK1 phosphorylation is lost thereby enhancing ULK1 kinase activity. In turn, ULK1 phosphorylates FIP200 and ATG13 to activate the complex^41,46,47^ (Fig. 2). The activated ULK1 complex translocates to the phagophore to initiate autophagosome formation.^42,48^ In a series of experiments aimed at defining the role of C9orf72 in autophagy initiation we found that depletion of C9orf72 did not affect the ULK1 complex at the level of mTOR, but rather prevented the translocation of the activated ULK1 complex to the phagophore.^39^

### C9orf72 as a Rab1a effector

In yeast the translocation of Atg1, the yeast homolog of ULK1, is regulated by Ypt1, the yeast homolog of Rab1^49^ and in mammalian cells Rab1 has been shown to regulate the early stages of autophagy.^50-52^ We therefore investigated whether the C9orf72-mediated regulation of ULK1 complex translocation involved Rab1. In cells depleted of Rab1a by siRNA treatment or expressing constitutively GDP-bound, dominant negative Rab1a(S25N), overexpression of C9orf72 no longer induced translocation of the ULK1 initiation complex and autophagy,^39^ indicating C9orf72 acted through Rab1a.

The structural homology of C9orf72 with DENN family Rab GEF proteins^30,31^ and the identification of C9orf72 as a potential Rab1a interaction partner in a yeast 2 hybrid screen^39^ prompted us to further investigate the interaction of C9orf72 and Rab1a in immunoprecipitation and *in-vitro* binding assays. Rab1a interacted with C9orf72 in both assays, but interestingly C9orf72 preferentially bound to GTP-bound Rab1a.^39^ Thus our data identified C9orf72 as an effector of Rab1a rather than a GEF as would be expected for a DENN family protein. As a Rab1a effector, C9orf72 was found to mediate the interaction between Rab1a and the ULK1 initiation complex and proved essential for Rab1a-mediated translocation of the ULK1 complex.^39^ Thus our data support a model in which C9orf72 is an effector of Rab1a that facilitates trafficking of the ULK1 initiation complex to the phagophore (Fig. 2).

### C9orf72 acts in a Rab cascade

While our work shows that C9orf72 is a Rab1a effector,^39^ others have shown that C9orf72 forms a complex with SMCR8 and WDR41^28,40,53,54^ and acts as a GEF for RAB8a and RAB39b in the autophagy pathway.^40^ However, the interaction between the C9orf72/SMCR8/WDR41 complex and RAB8a or RAB39b appeared to be mainly through binding with SMCR8, and Rab GEF activity was only observed when SMCR8 was present.^40^ As SMCR8 is itself a DENN domain protein,^31^ it appears that SMCR8 rather than C9orf72 may mediate the GEF activity toward Rab8a and Rab39b.

**Figure 3.**
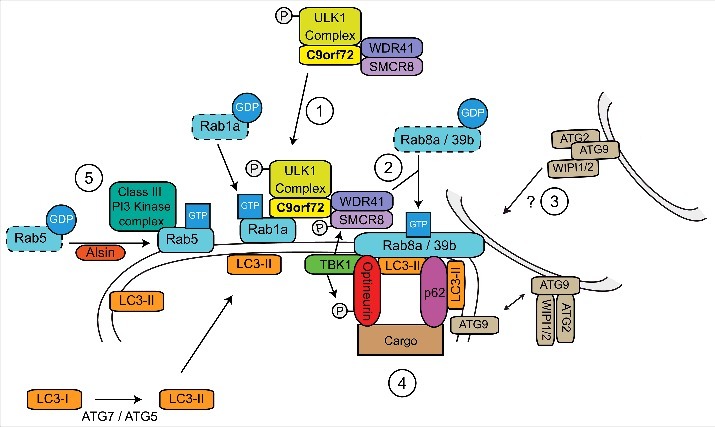
C9orf72 is a central hub in a Rab cascade pathway during autophagy induction. 1) C9orf72 controls the Rab1a-dependent translocation of the ULK1 initiation complex to the site of phagophore formation by interacting with the ULK1 complex and active GTP-Rab1a. 2) At the site of phagophore formation, the C9orf72/SMCR8/WDR41 complex functions as a GEF for Rab8a and Rab39b. 3) Active Rab8a and Rab39b may be involved in delivery of additional membrane to the site of phagophore formation by retrieval of ATG9 positive membranes. Additional membrane allows elongation of the nascent phagophore and formation of an autophagosome. 4) The autophagy receptors p62/sequestosome-1 and optineurin interact with Rab8a and Rab39b, promoting nucleation and site specific recruitment of autophagic substrates to the growing phagophore. Furthermore, Rab8a also recruits TBK1, leading to phosphorylation of optineurin, SMCR8 and ULK1. The phosphorylation of optineurin enhances its interaction with LC3-II, facilitating substrate delivery and the progression of autophagy. 5) The Class III PI3 Kinase complex is also required for autophagosome formation. Rab5, activated by Alsin, delivers the PI3 Kinase complex to the phagophore and also facilitates the recruitment of the ATG7-ATG5 conjugation system required for LC3-II formation.

Sequential activation of Rab GTPases in a cascade-like fashion allows the correct spatial and temporal recruitment of successive Rabs in a signal transduction pathway. In these Rab cascades the upstream Rab and its effectors recruit the GEF of the next downstream Rab, while the downstream Rab recruits the GAP for the upstream Rab (reviewed in ref. 55). Thus a model emerges in which C9orf72 links autophagy initiation to downstream events via a Rab cascade, involving Rab1a upstream of Rab8a and Rab39b (Fig. 3). Consistent with this, Rab1 has been shown to be involved in autophagy initiation^39,50,52^ whereas Rab8 and Rab39 are involved in autophagosome maturation^56,57^ (Fig. 1). Furthermore Rab8a and Rab39b were shown to interact with the autophagy receptors optineurin and p62/sequestosome-1 suggesting the interesting possibility of C9orf72 regulated site-directed autophagy initiation.^40,56^

### The role of C9orf72 in inflammation

Autophagy plays an important role in both innate and adaptive immunity. Thus, autophagy eliminates invading pathogens, controls pro-inflammatory signaling, regulates the secretion of immune mediators, and modulates MHC class II antigen presentation (reviewed in refs. 58, 59, 60).

*C9orf72* knockout mice present with varying degrees of autoimmunity and inflammation, accompanied by splenomegaly and increased expression of inflammatory cytokines.^23,25-29^ Our study into the regulation of autophagy initiation by C9orf72 indicates that these phenotypes could be a consequence of compromised autophagy. In support of this both *ULK1* and *AMPK*α (an upstream regulator of ULK1) knockout mice show splenomegaly similar to the *C9orf72* knockout mice.^61,62^ Furthermore, *C9orf72* knockout mice show increased secretion of IL-1ß^23^ and increased numbers of activated CD69^+^ T-cells,^25^ phenotypes that have previously been reported in autophagy-deficient *ATG16L* knockout mice^63^ and *ATG5* knockout mice,^64^ respectively. Hence the phenotype of these *C9orf72* knockout mice may relate to a specific role of C9orf72 in immune-related autophagy.

In agreement, TANK-binding kinase 1 (TBK1), a well-known regulator of inflammation and autophagy (reviewed in ref. 65) has been shown to phosphorylate SMCR8 in the C9orf72/SMCR8/WDR41 complex and this phosphorylation appears to be important for its function in autophagy.^40^ Furthermore, the C9orf72/SMCR8/WDR41 complex has been shown to interact with optineurin, an autophagy substrate receptor involved in clearance of intracellular bacteria and itself a TBK1 substrate.^40,66^ As mutations in both TBK1 and optineurin have also been associated with ALS/FTD,^67,68^ a pattern of disrupted immune-related autophagy linked to the pathogenesis of ALS/FTD is emerging.

### Defective autophagy and C9ALS/FTD

C9ALS/FTD patients show specific p62/sequestosome-1 pathology.^16,35,36^ To directly test whether this C9ALS/FTD-associated pathology was linked to *C9orf72* haploinsufficiency we investigated p62/sequestosome-1 distribution in cells and neurons depleted of C9orf72. Loss of C9orf72 resulted in the accumulation of p62/sequestosome-1 positive puncta in both HeLa cells and rat cortical neurons and this was specific to the loss of C9orf72 as reintroduction of C9orf72 in the rat cortical neurons was sufficient to rescue this accumulation.^39^ In a similar experiment, Sellier et al. also demonstrated that reduced expression of C9orf72 led to the accumulation of p62/sequestosome-1.^40^ We next went on to investigate autophagy in C9ALS/FTD patient-derived iNeurons and found markedly decreased levels of basal autophagy compared to their age and gender matched controls, lending further support to C9orf72 haploinsufficiency as a cause of autophagy deficits and p62/sequestosome-1 pathology in C9ALS/FTD.^39^ If loss of C9orf72 causes p62/sequestosome-1 pathology *in vivo* is not yet clear as p62/sequestosome-1 levels have not yet been reported in full or neuronal specific C9orf72 knockout mice.^25-29^ Interestingly neuronal specific ablation of C9orf72 did not lead to the accumulation of ubiquitin-positive inclusions in the spinal cord, hippocampus and frontal cortex, suggesting that at least in this case loss of neuronal C9orf72 is not enough to replicate disease associated ubiquitin pathology.^29^ It will be of interest to further characterize the role of C9orf72 in autophagy in the CNS to establish its relationship with p62/sequestosome-1 and ubiquitin pathology *in vivo*.

The data from *C9orf72* knockout mice show that loss of C9orf72 does not lead to the overt neurodegeneration that is typically associated with ALS/FTD.^23,25-29^ Similarly, GGGGCC repeat C9orf72 BAC-transgenic mice that model toxic gain-of-function RNA and DPR pathology (but retain mouse C9orf72) show no^24,69^ or mild neurodegeneration.^21,22^ Hence it is plausible that C9orf72 haploinsufficiency may be a modifier of GGGGCC repeat-associated toxic gain-of-function mechanisms. An attractive possibility is a 2-hit model in which reduced autophagy by loss of C9orf72 function exacerbates DPR accumulation and toxicity. Alternatively, defective autophagy caused by *C9orf72* haploinsufficiency in microglia and macrophages may lead to a failure in the support, and therefore survival, of (motor) neurons. Indeed, we know from studies of mutant SOD1-related ALS that ALS is a non-cell-autonomous process that critically involves glial cells.^70,71^ Furthermore, the association of C9orf72 with immune-related autophagy discussed above may provide a direct link to the neuroinflammation that is observed in ALS/FTD. Future experiments should dissect any direct pathogenic effects of immune-related autophagy in ALS/FTD by, for example, genetic inhibition of autophagy in specific cell lineages such as myeloid cells.

## Conclusions

**Figure 4.**
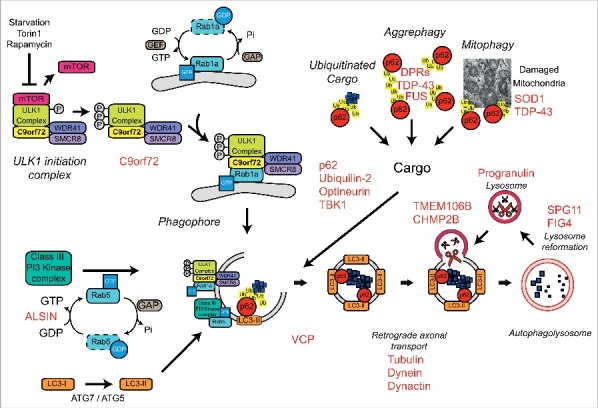
Autophagy and ALS/FTD. A number of genes, indicated in red, implicated in ALS/FTD have been linked to the autophagy/lysosomal pathway.

Autophagy deficits appear to have a central role in ALS/FTD. Indeed, in addition to C9orf72, TBK1 and optineurin many other genes implicated in the autophagy/lysosomal pathway have been associated with ALS/FTD, including Alsin (a Rab5 GEF), charged multivesicular body protein-2B (CHMP2B), p62/sequestosome-1, progranulin, ubiquilin-2, valosin-containing protein (VCP), Fig4, and TMEM106b (Fig. 4). Future research will have to elucidate the contribution of autophagy to the disease process and determine the effect of disease-associated mutations in neuronal and non-neuronal cells. Furthermore, as emerging evidence implicates multiple Rab GTPases in ALS/FTD and other neurodegenerative diseases, such as Parkinson disease, studying the specific functions of Rab GTPases in the CNS is warranted.
